# What Is Important at the End of Life? Perspectives From Experienced Home Care Workers

**DOI:** 10.1093/geroni/igaa057.230

**Published:** 2020-12-16

**Authors:** Emma Tsui, Emily Franzosa, Kathrin Boerner

**Affiliations:** 1 CUNY Graduate School of Public Health & Health Policy, New York, New York, United States; 2 James J. Peters VA Medical Center, Bronx, New York, United States; 3 University of Massachusetts Boston, Boston, Massachusetts, United States

## Abstract

Home care workers (HCWs) make up a large and rapidly growing sector of the American health care workforce serving older adults. This study focuses on a common but understudied feature of home care labor: workers’ thoughts around what makes a "good" or "bad" patient death. While researchers have investigated patients’, families’, physicians’, and other care providers’ perspectives on this issue, the perspectives of HCWs, who contribute substantially to home-based care at the end of life, have yet to be explored. We conducted 40 in-depth interviews with HCWs in New York City on their experiences with and reflections on patient death. We used a inductive, iterative approach to analyze data on what HCWs believe is important for dying patients. HCWs described EOL values that align well with the views held in common by patients, families, and other care providers, like the importance of not being alone when dying and being physically comfortable (not in pain and not suffering). In particular, HCWs conceptualized a detailed role for themselves when providing EOL care near the time of death. HCWs’ sustained presence and relationships with patients may uniquely position them to assist in the attainment of patients’ EOL goals, particularly when HCWs understand what these goals are. HCWs’ potential for playing this role, however, is jeopardized by a lack of training in EOL care and by the limited information they receive about a patient’s health status.

